# The prevalence of fear avoidance and pain catastrophising amongst patients with chronic neck pain

**DOI:** 10.4102/sajp.v76i1.1326

**Published:** 2020-01-29

**Authors:** Clare Cresswell, Mary L. Galantino, Hellen Myezwa

**Affiliations:** 1 Department of Physiotherapy, University of the Witwatersrand, Johannesburg, South Africa; 2 School of Health Sciences, Stockton University, Galloway, United States

**Keywords:** prevalence, fear avoidance, pain catastrophising, chronic neck pain, South Africa

## Abstract

**Background:**

Cognitive factors impact chronic pain, but the prevalence of fear avoidance (FA) and pain catastrophising (PC) in individuals suffering from chronic neck pain (CNP) has not been investigated in South Africa.

**Objectives:**

To determine the prevalence of FA and PC in patients with CNP at private physiotherapy practices in Johannesburg.

**Method:**

The Tampa Scale for Kinesiophobia-11 (TSK-11) (*α* = 0.80) and Pain Catastrophising Scale (*α* = 0.87) self-report questionnaires were used in a cross-sectional study to determine the prevalence of FA and PC, respectively. Descriptive statistics and correlations using Pearson’s or Spearman’s coefficient were conducted between demographic variables and FA and PC. Non-parametric data were tested using the Wilcoxon rank-sum or Kruskal–Wallis test. Cohen’s *d*-value or *r*-value measured strength of associations.

**Results:**

A sample of 106 CNP patients with a mean age of 48.7 years (± 14.8) from 25 randomly selected private practices participated in the study. Of the participants, 81% were women (*n* = 86). Fear avoidance and PC had a prevalence of 25.5% (*n* = 27) and 15.1% (*n* = 16), respectively. A positive correlation was found between FA-11-Total and PC-Total (*r* = 0.684; *p* = 0.0001) and between FA (TSK-11-Total and TSK-SF (somatic focus)) and PC and its subscales (*r* ≥ 0.602; *p* = 0.0001). Participants with a secondary education (26.0 ± 3.4) showed a higher FA than those with tertiary education (21.9 ± 1.5). Effect size was moderate (Cohen’s *d* = 0.60). Pain intensity correlated positively with both FA (Pearson’s correlation: *r* = 0.33; *p* = 0.001) and PC (Spearman’s correlation; *r* = 0.39; *p* = 0.0001).

**Conclusion:**

FA and PC affect a number of patients with CNP. A lower level of education was associated with FA and a higher pain intensity was associated with higher FA and PC.

**Clinical implications:**

Identifying FA and PC in patients with CNP is important to facilitate holistic management.

## Introduction

Chronic neck pain (CNP), with resultant disability, is common in the general population (Carroll et al. 2008), with studies showing that between 50% and 85% of those in pain are likely to experience recurring episodes of neck pain (Haldeman et al. 2010). It is reported that the cumulative incidence of having neck pain in early adulthood is high, as children and adolescents frequently experience neck pain and these symptoms tend to recur (Hoy et al. 2010). A considerable proportion of individuals never fully recover from their neck pain and, of patients suffering from neck pain, only 6.3% consider their pain as chronic (Côté et al. 2004).

Furthermore, persistent and/or chronic pain has been defined as continuous pain for at least three of the previous 6 months (Siddall & Cousins 2004). Different pain syndromes can be viewed as having their origins predominantly in a cognitive and affective environment as a result of a motor mechanism, an autonomic mechanism, a combination of some of them or all of them (Bolay & Moskowitz 2002).

While the cause of non-specific neck pain is unknown, the reasons for the onset and recurrence of neck pain have been recognised as multifactorial. Although we do not know the exact mechanism whereby pain starts, influences may or may not be modifiable and because of personal and environmental factors, others may be related to ‘occupation, headaches, emotional problems, low job satisfaction, sedentary work postures, a poor physical work environment (e.g., poor keyboard or mouse position), ethnicity and smoking’ (Hoy et al. 2010:789). Some studies suggest that CNP is more common in women than in men (Carroll et al. 2008). Sleep deprivation or disruption, anxiety and depression have also been associated with CNP, both as predictive factors and as a consequence of the condition (Elbinoune et al. 2016).

Cognitive factors as defined by Roy (2013:447–448) refer to ‘characteristics of the person that affect performance and learning. These factors serve to modulate performance such that it may improve or decline’ and have been found to be associated with disability, poorer outcome and higher levels of pain in patients suffering from CNP. Two cognitive factors include fear avoidance (FA) and pain catastrophising (PC) (Thompson, Oldham & Woby 2016). Vlaeyen et al. (2016:1588) developed the FA model and described fear as ‘the anticipatory emotional response to imminent threat’, and as a result of observation, experience or verbal instructions, rapid adaptive learning takes place. The updated FA model suggests that a patient gives pain, rather than ‘valued life goals’, priority and is likely to avoid rather than confront a painful stimulus or situation because of negative effects and harm of representation (Vlaeyen et al. 2016). The original iteration of the model suggested a sequential development of disability beginning with catastrophising to fear. However, analysis by a systematic review found that PC was not a mediator of disability in neck pain, while the effects of behavioural and physical treatment on disability may be mediated by catastrophising (Lee et al. 2015).

Pain catastrophising has been described as ‘an exaggerated negative “mental set” brought to bear during actual or anticipated pain experience’ (Sullivan et al. 2001:52). The FA model provides a rationale for relevant behaviours and outcomes when patients experience chronic pain (Leeuw et al. 2007; Waugh, Byrne & Nicholas 2014). Physiotherapists and other healthcare professionals addressing chronic musculoskeletal complaints have used this model to describe their approaches, treatment and management in this context (Lamé et al. 2005). Furthermore, in a recent study, Thompson and Woby (2018) have shown that targeting PC is particularly important when treating CNP. Haldeman (2010:425) also recognised ‘previous neck injury, high pain intensity, self-perceived poor general health, worrying, getting angry or frustrated’ as contributors to recurring neck pain.

In a South African context, various studies have determined the prevalence of chronic pain to be between 36.3% and 42.9% in primary health and rural settings, the most common body regions being the back, knee, ankles, head and shoulders (Igumbor et al. 2011; Rauf et al. 2013). And more specifically, a prevalence of 36% was found in patients suffering from chronic musculoskeletal pain in a primary health clinic in Cape Town (Parker & Jelsma 2010). The prevalence of FA and PC related to CNP has not been established in South Africa. However, both factors have been found to be present in workers who suffer from low back pain and work in the South African steel manganese industries (Van Vuuren et al. 2005, 2006).

Studies highlight the significance of FA and PC in patients who experience low back pain, osteoarthritis and anterior knee pain (Domenech et al. 2013). Recovery from neck and shoulder pain has been shown to be impacted by FA beliefs and PC (Karlsson et al. 2016). When clinicians help patients acquire pain knowledge, catastrophising may be reduced and pain and function are improved with increased pain knowledge (Lee et al. 2016). The complexity of co-morbidities associated with CNP shows that involving a multidisciplinary health team should be considered when planning treatment and symptom management for these individuals.

Numerous studies undertaken over the past two decades show that FA and PC impact patients’ pain and disability as chronicity emerges. In order to add to the body of knowledge, particularly from the perspective of a middle- to low-income country, we chose to select our participants from private physiotherapy practices as CNP is a common condition encountered by practitioners. The purpose of our study was to establish the extent of FA and PC in a group of South African patients who have CNP and to understand the factors influencing these two debilitating symptoms.

## Methods

A cross-sectional study used two validated self-report questionnaires to investigate the prevalence of FA (Woby et al. 2005) and PC (Sullivan et al. 1995) in patients suffering from CNP for longer than 3 months. Patients suffering from CNP were invited to participate in this study when they attended physiotherapy at a private practice in the Johannesburg area.

Physiotherapy practices offering musculoskeletal treatment in Johannesburg, affiliated with the Orthopaedic Manipulative Physiotherapy Group (OMPTG) of the South African Society of Physiotherapy, were selected from the OMPTG website after getting permission from the chairman of the organisation. The names of the practices were then randomly selected using the Excel Randomisation Programme. Each practice owner was approached sequentially to identify their willingness to participate in the study. In their turn, the physiotherapists at these clinics identified willing participants based on our inclusion and exclusion criteria and enrolled them sequentially.

### Sample size

The estimated size of the sample was based on the proportion of patients with symptoms of FA and/or PC. An estimate of 50% prevalence was used, with 10% precision, a 95% confidence level and an infinite population. Thus, a minimum sample size of 97 patients with CNP was required (Daniel 1999).

### Ethical considerations

The study was approved by the Human Research Ethics Committee (Medical) of the University of the Witwatersrand (Clearance Certificate Number M140434). Informed consent forms were signed by all the participants.

### Procedure

**FIGURE 1:**
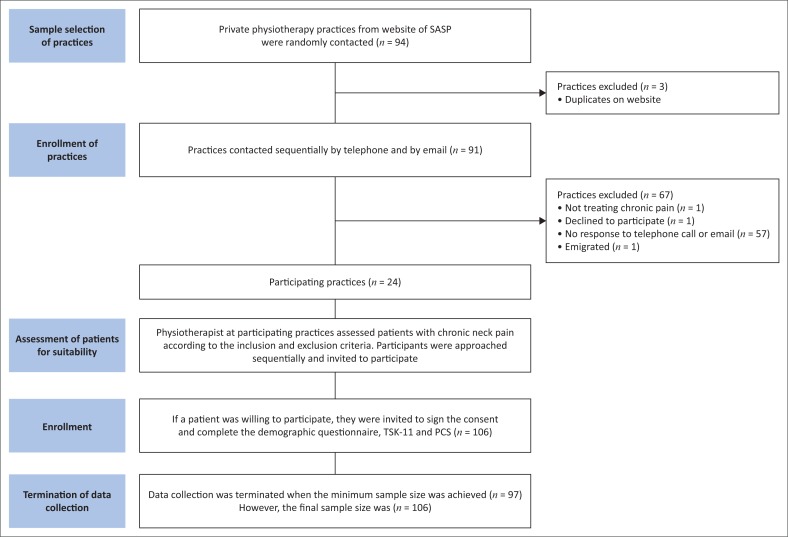
Flow diagram showing the sampling procedure of practices and participants.

A summary of the study procedure is presented in Figure 1. After physiotherapist review of patient history, the study was explained to the participants and approved informed consent was obtained. Then the participants completed three questionnaires. The practice retained a form with the participant’s name and the identifying code on each questionnaire to ensure that participants were not approached more than once.

Data were collected over a 16-month period between 2014 and 2015. The study’s inclusion criteria were as follows: participants suffering from neck pain for three or more months; the body areas for symptoms should include pain, muscle tension or stiffness in the neck, shoulder area, upper back area and/or above the costal margin, with or without pain in the arms; participants’ aged 18 years or older; and participants undergoing or had previously received physiotherapy. No participant was excluded based on gender, ethnicity or nationality.

The exclusion criteria for the study were as follows: if participants showed signs of neurological impairment from nerve compression (as this might constitute a more acute and specific neck condition), had a progressive neuromuscular condition and unexplained weight loss, received drugs via intravenous means or had undergone any surgical intervention in the previous 3 months.

### Study variables and questionnaires

All participants completed a demographic survey and the English versions of two validated self-report questionnaires that screened for FA (Tampa Scale for Kinesiophobia-11 [TSK-11]) and PC (Pain Catastrophising Scale [PCS]). Demographic data included gender, age, marital status, highest level of education and employment status. All the questionnaires were completed by the participants, although they could ask the physiotherapist if they needed clarification.

The impact of pain was determined by noting the duration of neck pain in months, and if the participants’ ability to work had been reduced. Pain intensity for each participant at the time of questionnaire collection (current) was measured by means of a 10-cm Visual Analogue Scale (VAS) (where 0 represents ‘no pain’ and 10 represents ‘the worst pain imaginable’) to denote the pain experience ‘now’ (Nordin et al. 2009). Boonstra et al. (2008) investigated the reliability and validity of the VAS for chronic pain patients and found that *p*-values varied between 0.60 and 0.77 in a reliability study and between 0.76 and 0.84 in a validity study (Boonstra et al. 2008).

The TSK-11 is used to measure FA and it includes a somatic focus (SF) subscore, an activity avoidance (AA) subscore and a total score. The scale includes 11 items, each with a range from 1 to 4. The TSK-11 total score ranges from 11 to 44, *α* = 0.79 (Woby et al. 2005); the TSK-11-SF subscore ranges from 5 to 20, *α* = 0.68; and the TSK-11-AA subscore ranges from 6 to 24, *α* = 0.67 (Roelofs et al. 2011). While the TSK-11 questionnaire has been validated in English, it has not yet been translated into any other South African languages.

There are no cut-off scores for clinically relevant levels of FA for the TSK-11. The term ‘clinical relevance’ refers to the level where behaviour becomes important in a clinical setting and is frequently used in psychological literature where there is no definite ‘cut-off’ score for a particular behaviour (Sullivan et al. 1995). However, in the TSK-17 (the form of the TSK as described by Vlaeyen et al. [1995]), the median score was considered a cut-off point, indicating that those who scored higher than 37 would be considered to be high avoiders, while those who scored lower than 37 would be considered to be low avoiders (Vlaeyen et al. 1995). As a shortened form of the TSK-17, the validated TSK-11 proved to be useful for the sake of brevity (Woby et al. 2005). For the purposes of our study, the scores above the midpoint of each scale were taken to indicate that the participant would be likely to show signs of greater FA.

The PCS is summarised as a total score of 13 items, each ranging from 0 to 4, and consisting of the sum of three subscales – rumination (PCS-R), helplessness (PCS-H) and magnification (PCS-M). A high internal consistency was established: *α* = 0.66–0.87 (Osman et al. 2000; Sullivan et al. 1995). We chose the cut-off score of greater than or equal to 30 as it is considered to indicate high catastrophisers (Sullivan et al. 1995). The PCS questionnaire has been translated into 20 languages and has been validated for use amongst patients with fibromyalgia who speak South African English, Afrikaans and Xhosa (Morris et al. 2012).

### Statistical analysis of the data

The prevalence of patients with clinically relevant levels of FA and PC was calculated by dichotomising the PCS and TSK-11 scales and subscales, using their cut-off and midpoint scores, respectively. Cronbach’s alpha was determined for the TSK and PCS scales, as well as their subscales. The relationship between PC (PCS scales and subscales) and FA (TSK scales and subscales) was assessed by the Spearman rank-order correlation coefficient because the PCS data were not normally distributed. The strength of the associations was measured by interpreting the absolute values of the correlation coefficients (Cohen 1988).

The relationships between the scores for the respective categories of the demographic variables used in the study and each of the PCS and TSK main scales were assessed by means of an unpaired *t*-test (or analysis of variance [ANOVA] in the case of more than two groups). Where the data did not meet the assumptions of these tests, a non-parametric alternative, the Wilcoxon rank-sum test (or the Kruskal–Wallis test for more than two groups) was used. The strength of the associations was measured by means of a Cohen’s *d*-value for parametric tests and an *r*-value for non-parametric tests (Cohen 1988). Depending on the distribution of the data, the relationships between the continuous demographic variables and the scores for each of the PCS and TSK main scales were assessed by Pearson’s or Spearman’s correlation coefficient (Cohen 1988). STATISTICA, version 12 (StatSoft, Inc. 2013, www.statsoft.com), was used for data analysis and the 5% significance level was applied.

## Results

Of the 94 practices in our randomised list, 24 participated in the study. Three practices were excluded from the list as they were duplicates.

During this process, 67 practices were excluded for the following reasons: practices did not treat chronic pain conditions (*n* = 1), declined to participate (*n* = 8), had no response to telephone call or e-mail (*n* = 57) and had closed because the owner had emigrated (*n* = 1). The participating practices were situated within a 30-km radius of central Johannesburg (Figure 1).

### Demographic information

The study sample comprised 106 participants with CNP. Table 1 presents the demographic information of the participants. Of the participants, the majority were women (81.1%), with a mean age of 48.7 years; 67.9% were married or in a relationship and had a tertiary education (76.4%). Most were working full-time (56.6%), with an even distribution for those working part-time, unemployed or retired. Details of occupation were not requested in the demographic data collection, and most had not reduced their work load because of their pain (79.2%).

**TABLE 1:** The demographic profile of the participants (*n* = 106).

Variable	*n*	%	Mean (SD)/Median (interquartile range)	Range
Overall number	106	-	-	-
**Gender**				
Female	86	81.1	-	-
Male	20	18.9	-	-
Age (years)	-	-	48.7 ±14.8	20–80
Pain intensity (VAS)	-	-	4.4 ±2.2	0.3–8.7
Pain duration (years)	-	-	8 (2.5–15)	0.25–63
**Highest level of education**				
Primary	1	0.9	-	-
Secondary	22	20.8	-	-
Tertiary	81	76.4	-	-
Unknown	2	1.9	-	-
**Employment status**				
Full-time	60	56.6	-	-
Part-time	21	19.8	-	-
Retired/unemployed	23	21.7	-	-
Student	1	0.90	-	-
Unknown	1	0.9	-	-
**Marital status**				
In a relationship	72	67.9	-	-
Single	34	32.1	-	-
**Reduced work load because of pain**				
No	84	79.2	-	-
Yes	14	13.2	-	-
Unknown	8	7.5	-	-

SD, standard deviation; VAS, Visual Analogue Scale.

The mean pain intensity was 4.4 (±2.2) and the median pain duration was 8 years.

### Analysis of Tampa Scale for Kinesiophobia-11 and Pain Catastrophising scales and their subscales

The mean scores for the TSK-Total, the TSK-SF (SF) and the TSK-AA (AA), and the median scores for the PCS and its subscales – PCS-R, PCS-H and PCS-M – are provided in Tables 2 and 3, respectively. The data for PC were concentrated around the lower end of all four scales, with relatively few patients reporting very high levels.

**TABLE 2:** Scores obtained for the Tampa Scale for Kinesiophobia-11 scale and its subscales (*n* = 106).

Scale	Mean	SD	Interquartile range		*α*	Cut-off for this study (scale midpoint)	Prevalence of TSK (%)	95% confidence interval for TSK (%)
TSK-11-total	22.9	7.0	17.0	28.0	0.85	> = 28	25.5	18.1–34.5
TSK-SF	9.8	3.6	7.0	12.0	0.75	> = 13	24.5	17.3–33.5
TSK-AA	13.0	4.1	10.0	16.0	0.77	> = 16	25.5	18.1–34.5

*α*, Cronbach’s alpha; TSK, Tampa Scale for Kinesiophobia; SF, somatic focus; AA, activity avoidance; SD, standard deviation.

**TABLE 3:** Scores obtained for the Pain Catastrophising Scale and its subscales (*n* = 106).

Scale	Median	SD	Interquartile range		*α*	Cut-off for this study	Prevalence of PCS (%)	95% confidence interval for PCS (%)
PCS-total	12	11.9	6.0	22.0	0.95	≥ 30	15.1	9.5–23.1
PCS-R	5	4.6	2.0	8.0	0.93	> 11	14.2	8.8–22.0
PCS-M	2	2.5	1.0	4.0	0.75	> 5	23.6	16.5–32.5
PCS-H	5	5.7	2.0	11.0	0.91	> 13	17.9	11.8–26.3

PCS, Pain Catastrophising Scale; R, rumination; M, magnification; H, helplessness; SD, standard deviation.

The reliability of the TSK-11 and the PCS scales and their subscales, as measured by Cronbach’s alpha, was more than 0.70, exceeding the available values as recorded in the literature (Roelofs et al. 2007; Sullivan et al. 1995; Woby et al. 2005).

The prevalence of patients with clinically relevant levels of FA was between 24.5% and 25.5% for all three scales (Table 2). The prevalence of participants with clinically relevant levels of PC ranged between 14.2% and 23.6% (Table 3). Differences in the prevalence of participants above the cut-off scores in the subscales of both the TSK-11 and PCS were not statistically significant.

### Association between fear avoidance and pain catastrophising

All correlations between the TSK-11 and PCS scales and subscales were significant and all corresponded to large effect sizes (*r* ≥ 0.50) (Table 4). The correlation coefficients were moderately high, indicating that FA (the TSK score) increases as PC (the PCS score) increases.

**TABLE 4:** Correlation between the Tampa Scale for Kinesiophobia-11 and Pain Catastrophising scales and their subscales.

Variable	Spearman rank-order correlation coefficients			
	PCS-total	PCS-R	PCS-M	PCS-H
TSK-11-total	0.684*	0.624*	0.653*	0.657*
TSK-SF	0.684*	0.608*	0.602*	0.678*
TSK-AA	0.548*	0.499*	0.576*	0.517*

TSK, Tampa Scale for Kinesiophobia; PCS, Pain Catastrophising Scale; R, rumination; M, magnification; H, helplessness; SF, somatic focus; AA, activity avoidance.

* , *p* < 0.001.

### Association between the demographic variables, pain intensity, fear avoidance and pain catastrophising

The association between demographic variables, pain intensity and the two cognitive factors under investigation, namely FA and PC, was analysed and the following significant results were found. The mean FA (TSK-11) score for those patients with a primary and secondary education (26.0 ± 3.4) was higher than that for those with a tertiary education (21.9 ± 1.5) (*p* = 0.013; moderate effect size: Cohen’s *d* = 0.60), indicating that those with a primary and secondary education were more likely to be fear avoidant than those with a tertiary education. No significant association was found between the highest level of education and PC.

A significant positive correlation, although low, was found between FA and pain intensity (TSK-11 score [*r* = 0.33; *p* = 0.001]). Similarly, a significant positive low correlation between PC and pain intensity was found (PCS score [*r* = 0.39; *p* < 0.0001]). The effect size was low in both cases. The positive correlation in both cases indicates that as FA and PC increase, so does the pain intensity. However, no significant associations were found between gender, age, employment status, marital status, reduced work load because of pain or pain duration, and the respective TSK and PCS scores.

## Discussion

The prevalence of FA was found amongst one quarter of the sample participants, while the prevalence of PC was found amongst one-sixth of the participants. While the prevalence was relatively low, one in four or six patients who present for treatment with CNP could have these cognitive factors, which may impact their recovery. Conversely, as only a relatively small proportion of patients with CNP were fear avoidant or had catastrophic thoughts, it is important to recognise that long-term CNP is not always associated with cognitive factors. No studies have investigated the prevalence of FA or PC in chronic non-specific neck pain in a South African context. While studies have shown the prevalence of 5.2% for patients with CNP and a history of previous trauma to the cervical spine, we chose not to identify this subgroup (Guez et al. 2002).

Fear avoidance responses vary and the evidence suggests that those who have task-specific chronic pain may differ from others who have generalised pain disorders (e.g. fibromyalgia), or who believe that their pain cannot be controlled (Meulders, Vansteenwegen & Vlaeyen 2011). We did not request diagnoses from participants, so we were unable to make any deductions. Although 25% of our participants experienced FA, a considerable number of participants did not avoid movement, and our findings showed that the duration of pain was not associated with either PC or FA.

Opinions vary as to whether FA or PC accounts for increased pain and disability (Thompson et al. 2010); however, a study found that greater PC, rather than FA, accounts for an increase in pain intensity and disability in chronic non-specific neck pain (Thompson & Woby 2018). Despite the many factors that have been shown to have a disabling impact on patients with CNP, leisure and work habits are most likely to be responsible for their lack of physical activity (Mansfield et al. 2018). We did not request this information from our participants. Furthermore, patients with high levels of FA are more likely to experience high pain intensity levels (Vaegter et al. 2018) and our study confirms these findings.

Fear avoidance beliefs and PC have mediating effects on pain intensity in post-traumatic whiplash injuries (Andersen 2016), and a recent study on chronic non-specific neck pain suggested that while changes in levels of pain intensity are important in influencing the level of disability, a reduction in PC is particularly important (Thompson & Woby 2018). Measurement of pain and appropriate interventions to address biological, psychological and social factors that in turn impact pain are important, and it is useful to assess PC and FA (Louw et al. 2016). The mean pain intensity was scored at 4.4 on the VAS in our study and is classified as moderate interference (Boonstra et al. 2014). The mean age of our participants was 48.7 years. Evidence suggests that compared with older or younger age groups, the 45–59-year age group is almost four times as likely to suffer chronic, recurrent or continuous neck pain and has the highest risk and poorest prognosis for recovery (Carroll et al. 2008). Most patients had suffered from neck pain for a number of years (median 8 years). This supports the findings of the persistent nature of chronic pain in other studies although, interestingly, no association was found between pain duration and either FA or PC in our study.

Additional studies do not show that pain duration as a health index is associated with pain reduction in treatment programmes, thus confirming the ongoing suffering of the individual from chronic pain (Severeijns et al. 2004). Continued suffering shows the importance of identifying at-risk patients during the acute stage so that steps can be taken to reduce factors that may contribute to the possible development of chronic pain (Carroll et al. 2008). However, both PC and FA or pain-related fear have been identified as risk factors for developing chronicity and disability and have been shown to mediate treatment efficacy (Verhagen et al. 2010). Therefore, it is important to identify risk factors including cognitive perspectives that may lead to FA and PC. Our results show that should a higher FA level manifest in patients, they would tend towards a higher level of PC, pointing to an increased concern with bodily harm or hypervigilance.

Fear avoidance beliefs may not limit all activities but may lead to limiting specific activities (Leonhardt et al. 2009). Although used for decades, FA and fear belief models may be too simplistic as explanations for the complexity of the chronic pain sufferer (Moseley & Vlaeyen 2015). When FA beliefs are analysed in the general population, no difference can be found between these and their prevalence in people with mild to moderate pain (Boonstra et al. 2014) and in those with no pain at all (Goubert, Crombez & De Bourdeaudhuij 2004). While some researchers suggest that fear of pain and PC before injury may lead to fear avoidant beliefs and behaviour, others propose that these cognitive factors may be the consequences of ongoing or persistent pain (Parr et al. 2012). Research into FA or PC seeks to establish whether patients are neurobiologically more susceptible to transitioning from acute to chronic pain through sensitisation. However, causal results are unclear (Linton et al. 2018).

Central nervous system changes occur when patients with chronic low back pain present with FA (Lamoth et al. 2004), although Malfliet et al. (2015) indicated that in chronic idiopathic neck pain, central sensitisation is not a characteristic feature. Maladaptive pain coping behaviours such as FA and PC are important aspects of the puzzle of chronic pain as are other co-morbidities, such as depression and sleep deprivation (Chou & Shekelle 2010). Because our study primarily investigated the prevalence of FA and PC, we were unable to include the impact of co-morbidities on these cognitive factors. However, more research is needed before we understand why some people develop chronic pain and not others so that strategies can reliably halt the process (Eccleston & Crombez 2017; Moseley & Vlaeyen 2015).

Our study tested selected demographic factors to find their association with FA and PC. A negative correlation was found between the level of education and that of FA, indicating that those with a primary and secondary education are more likely to be fear avoidant than those with a tertiary education. It is not clear how these results are important as a systematic review carried out by Chou and Shekelle (2010) showed that education level is not a strong predictor for developing chronic pain (Chou & Shekelle 2010). Furthermore, Jelsma, Mkoka and Amosun (2008) suggest that caution needs to be followed when associating socio-economic status factors, including education and employment, with psychosocial factors in a South African context. They posited that work status may not equate to the level of education, and therefore psychological risk factors, because economically disadvantaged people may be involved in informal work (Jelsma et al. 2008).

Interestingly, 79.2% of our participants had not reduced their workload as a result of pain, thus suggesting the intensity of their pain, FA and PC had not resulted in work-related disability. As our participants were recruited from physiotherapy practices, we are unable to say if their treatments had had any influence on their disability levels as many interventions, both physical and psychological, have been shown to modify PC in particular (Sullivan et al. 1995; Sullivan 2012).

This effect may be a factor that we did not take into consideration during our study. It has been shown that behavioural changes occur when certain aspects of the therapeutic relationship have been addressed. Wilson et al. (2017) noted that patients develop a greater self-awareness when the therapist works with ‘the whole’ of the patient and was ‘more than just a professional’, and that the patient was able to ‘work through challenges within the therapeutic relationship’ (Wilson et al. 2017:100). By identifying the cognitive factors that are associated with the more resistant and complex chronic pain sufferers, clinicians can develop their expertise in analysing the implications of avoidant and catastrophic responses as two of the wide range of psychosocial and behavioural factors that could impact a patient’s pain response (Keefe, Main & George 2018). Furthermore, working with patients with chronic pain to get them to understand the efficacy of self-management strategies will help these individuals manage their pain (Devan et al. 2018:384). Importantly, the possible strategies that may be helpful for self-management are:

Self-discovery – the ability to distinguish self (i.e., body, thoughts and feelings) from pain; feeling empowered by incorporating self-management strategies into practice; and supportive ambience via collaborative relationships with clinicians and support from family and friends.

Unhelpful strategies or barriers include ‘difficulty with sustaining motivation for pain self-management; distress experienced from ongoing pain, anxiety and depression; and unsupportive relationships with clinicians, family and friends’ (Devan et al. 2018:388).

The addition of psychologically informed physiotherapy to a physiotherapist’s armamentarium has meant that the psychosocial factors that impact a patient’s recovery can be addressed. These include graded activity, goal setting, problem solving, cognitive restructuring, pacing, relaxation training and education (Nijs et al. 2014). The education component is commonly known as pain neuroscience education (PNE). Together with a good therapeutic alliance, PNE has been shown to have a positive effect on pain perception, PC, disability and physical performance, which can help to address the cognitive factors identified by outcome measures such as FA and PC (Nijs et al. 2014).

Noteably, the challenge remains for clinicians to sustain improvements achieved by interventions as shown by a 10-year follow-up study where this was not the case (Emilson et al. 2017).

To date, no studies have been conducted on the effects of PNE for CNP in the South African context, but a systematic review by Louw et al. (2016) showed it to be effective in treating FA and PC in various conditions such as chronic fatigue syndrome (Meeus et al. 2010) and chronic pain (Gallagher et al. 2013). However, a Cochrane review in 2009 of patient education delivered to patients with both acute and CNP did not show clearly that there was significant benefit to the patient (Haines et al. 2009; Louw et al. 2016). The results of the systematic reviews are conflicting and further research, particularly in a South African context, into the content of the patient education delivered may give clarity as to the value of this treatment. Clinicians have a valuable role in thoroughly assessing patients by including relevant tools to determine whether cognitive factors impact their recovery. If an effective therapeutic alliance has been established and the patient has a solid understanding of pain neuroscience and the factors contributing to their condition, patients will be able to partner with the clinician to work towards optimal recovery.

## Limitations

Several limitations of our study are worth highlighting. The sample size was modest and additional associations between demographic variables and pain measures may become evident with a larger sample size. Additionally, other demographic data could have been included, such as how many recurrences patients had had over a period of time, as well as International Classification of Diseases diagnosis. Hoy et al. (2010) noted that the 1-year incidence of neck pain was between 10.4% and 21.3%, and our study did not capture these data. Furthermore, an understanding of the influence of sleep, depression and anxiety could have given important insights into CNP in these participants. It would have been interesting to investigate if participants had suffered a whiplash or any injury to their cervical spines as they are known to have a poorer outcome in the long term (Hoy et al. 2010). Our definition of CNP did not distinguish between those who had recurrent and/or episodic neck pain, with no symptoms in-between episodes, and those who were never free of neck pain. With a larger sample size, this may produce interesting results by asking participants if they had other areas of pain. Further demographic data, such as ascertaining if English was the participant’s first language, cultural groupings and the type of work, may have given more insights into FA and PC in a South African context. As the number of patients who declined to participate in the study was not determined, self-selection biases may be inadvertently inherent in our study.

Our study was conducted in the limited setting of private physiotherapy practices in Johannesburg where patients are able to pay for their healthcare. Thus, our results cannot be generalised to other populations. A considerable number of physiotherapists in the urban areas of South Africa work in private practice settings (*n* = 3288) and are utilised by upper- to middle-class populations (Diener 2016). The lower socio-economic sector, which represents a large proportion of the South African population, was not represented in this study and further research should include under-served physiotherapy services (*n* = 758 physiotherapists in public service, including students) (Diener 2016). Thus, further research is needed to address patients of different economic classes and language groups. It is important for patients to answer questionnaires in their primary language as some concepts are not translatable across various groups in South Africa (Mkoka et al. 2003; Parker, Jelsma & Stein 2016).

## Conclusion

This study provides valuable information about the prevalence of FA and PC affecting patients with CNP conditions in private physiotherapy settings in Johannesburg, South Africa. Our findings showed that FA and PC are present in a subset of patients undergoing physiotherapy. The level of education and pain intensity impacted these two cognitive factors in our study sample and the findings suggest that clinicians should tailor their treatment according to the presence of these cognitive and psychosocial factors to optimise patient outcomes. Further research is needed to address patients of different geographical areas, socio-economic classes and language groups.
